# Catalytic nanotherapeutics with cancer cell membrane and chitosan-coated Cu/Pt nanoparticles for gastric cancer precision therapy

**DOI:** 10.1186/s13036-026-00676-3

**Published:** 2026-05-09

**Authors:** Wanhong Zhang, Yuchao Ma, Linjie Li, Yiyu Shi

**Affiliations:** 1 Shanxi Province Cancer Hospital, Shanxi Hospital Affiliated to Cancer Hospital, Cancer Hospital, Chinese Academy of Medical Sciences, Shanxi Medical University, Taiyuan, 030013 China; 2 Central Laboratory, Shanxi Key Laboratory of Heart Failure Precision Medicine, Shanxi Cardiovascular Hospital, Cardiovascular Hospital Affiliated to Shanxi Medical University, No. 18 Yifen Street, Wanbailin District, Shanxi, 030024 China

**Keywords:** Cancer cell membrane, Gastric cancer, Cu/Pt NPs, Chitosan, Nanoplatform

## Abstract

Gastric cancer remains a major global health challenge, underscoring the need for highly effective and precisely targeted therapies. This study presents a novel biomimetic nanoplatform comprising cancer cell membrane-coated chitosan-stabilized Cu/Pt nanoparticles (CCM@Ch-Cu/PtNPs), designed to enhance tumor specificity and therapeutic efficacy. We characterized the nanocomposite using X-ray diffraction (XRD), X-ray absorption spectroscopy (XAS), and transmission electron microscopy (TEM), confirming its bimetallic architecture featuring Cu-rich and Pt-rich surface domains. In vitro, treatment with CCM@Ch-Cu/PtNPs (75 µg/mL) markedly decreased viability and colony formation in AGS and HGC gastric cancer cell lines, induced apoptosis, and triggered reactive oxygen species (ROS) generation, indicating potent cytotoxic activity and disruption of oncogenic pathways. In vivo, systemic administration of CCM@Ch-Cu/PtNPs in a murine gastric cancer model resulted in preferential tumor accumulation, substantial tumor growth inhibition, and prolonged survival, all achieved without detectable organ toxicity. These results support the potential of CCM@Ch-Cu/PtNPs as a theranostic nanomedicine platform for targeted gastric cancer therapy.

**Supplementary Information:**

The online version contains supplementary material available at 10.1186/s13036-026-00676-3.

## Introduction

Gastric cancer (GC) is one of the most common and fatal cancers, 40% of all instances and fatalities happen in China, which is dangerous for people’s health. Nearly 780,000 people lost their lives to GC, making it the sixth most common cancer in the world [1]. Since the MAGIC trial in 2006, the significance of perioperative chemotherapy in locally advanced gastric cancer has been well established [2]. The biological features primarily indicate a rise in ferrous ion content within the cell, along with an increase in reactive oxygen species (ROS) production and glutathione (GSH) consumption, leading to lipid peroxidation [3, 4]. The stomach is a multifaceted organ with several anatomical parts (antrum, body, and cardio) that contain various cell types and functions. A crucial phase in gastric cancer carcinogenesis is intestinal metaplasia (IM), a pre-malignant state in which the gastric epithelium is substituted by cells resembling the small intestine [5]. Chemotherapeutics aren’t very specific, they could harm healthy immune cells like macrophages organs like the digestive tract, bone marrow, and hair follicles. There is growing optimism about the potential of nanotechnology in healthcare. Advances in nanotechnology have resulted in the creation of biomedically useful nanomaterials [6]. Because of its impressive capabilities in extending the payload circulation period, decreasing interaction with macrophages, and improving homotypic target ability [7]. The platinum (Pt) nanoparticles enhance the performance of various enzyme mimics, including peroxidase, oxidase, and catalase. Curiously, Pt-based NPs exhibited P53-mediated growth inhibition in addition to DNA damage induction [8]. Due to its numerous functions and great biocompatibility, cationic polysaccharide chitosan (CS) a byproduct of N-deacetylation of chitin is a promising contender for use in biomedical applications. This polymer’s chemical structure makes it ideal for ligand functionalization, and thermo- and pH-responsive NP production [9]. For example, the plasmonic characteristics of Cu@Pt bimetallic core-shell NPs, greatly improve the efficiency of the light. One potential solution for effectively treating airborne contaminants is platinum nanoparticles, or Pt NPs [10]. The biomedical community extensively uses chitosan since it is a biodegradable, non-toxic polymer with strong anti-inflammatory and antioxidant properties and regulate its release rate [11]. One potential use for chitosan nanoparticles is to deliver chemotherapeutics to the tumor microenvironment in a way that is both more effective and less harmful to the cells [12]. The gastrogenesis continues to operate through the NUAK1/STAT5/GLI1 signaling axis. When patients with GC have an overexpression of NUAK1, it is a sign of a worse prognosis and a factor in chemoresistance. A previously reported study demonstrates that targeting NUAK1 provides a novel therapeutic approach for the treatment of GC, both on its own and in conjunction with existing chemotherapies [13]. Recent developments in immunotherapies such as PD-1/PD-L1 checkpoint blocking and CAR-T cell treatment have made great strides in treating gastric cancer [14]. Lee et al. (2021) showed that the gastric cell line AGS expressed factors involved in autophagy and apoptosis when exposed to oleanolic acid (OA). They also examined the relationship between autophagy and apoptosis. The relationship between cell deaths and the PI3, mTOR pathway was further investigated, and experiments were conducted to ascertain the In vivo effect [15]. To effectively eliminate circulating tumor cells (CTCs) in entire blood samples, Lin et al., 2024 detailed the creation of platinum prodrug delivery composites disguised as cancer cell membrane biomimetics. Utilizing cancer cell membranes enhances the effectiveness of homologous targeting while decreasing protein interference. Infused into the central nanoparticle were compounds of platinum (IV). So, without compromising therapeutic efficacy, it can increase inhibitory effects on cancer spread and decrease drug-related side effects [16]. Recently, the combination of anti-programmed cell death protein with immune checkpoint inhibition and chemotherapy in neoadjuvant therapy has demonstrated encouraging short-term oncologic outcomes in locally advanced gastric cancer [17]. In the present study, combining cancer cell membranes with nanoparticles is a promising strategy in cancer treatment. It enhances the specificity and effectiveness of drug delivery while minimizing systemic toxicity, making it a valuable tool in the fight against gastric cancer. We used cancer cell membrane-coated chitosan-Cu/Pt nanoparticles (CCM@Ch-Cu/PtNPs) to create a novel biomimetic nano platform for gastric cancer targeting. In vitro tests revealed that CCM@Ch-Cu/PtNPs decreased the viability of AGS and HGC cells, caused apoptosis, and released ROS. Nanoparticles selectively accumulate in tumor tissue, reducing tumor growth responses without organ toxicity. This study aims to comprehensively delineate the significance of the study within the broader framework of this emerging field.

## Materials and methods

### Synthesis of Cu/Pt nanoparticles

Bimetallic nanoparticles of Cu/Pt were produced using a solvothermal technique. Copper(II) acetylacetonate (Cu(acac)₂, ≥ 98% purity) and a platinum precursor (≥ 98% purity) were procured from Sigma-Aldrich and utilized without additional purification. To synthesize the nanoparticles, 0.026 g of copper precursor and 0.040 g of platinum precursor were dissolved in 10 mL of benzyl ether while being magnetically stirred. The precursor feeding ratio was determined during initial optimization studies to achieve an essentially equiatomic Cu/Pt content in the resultant nanoparticles. The reaction mixture was initially exposed to a vacuum at 95 °C for 1 h to eliminate residual moisture and dissolved gases. Thereafter, the system was evacuated with nitrogen and heated to the reaction temperature at a rate of 15 °C per minute. The solution was sustained at an increased temperature for one hour under continuous nitrogen protection to facilitate nucleation and alloy formation. Upon completion of the reaction, the mixture was progressively cooled to ambient temperature under nitrogen atmosphere. The resultant nanoparticles were obtained via centrifugation, subsequently washed multiple times with ethanol and hexane to eliminate unreacted precursors and surplus organic residues, and then dehydrated under vacuum for further analysis [18].

### Cancer cell membrane extraction (HCT116)

The manufacturer’s directions to isolate and fractionate cancer cell membranes using the isolation and cell fractionation kit for plasma membrane proteins (Invent Biotechnologies Cat. No. 10002). The technique guarantees total isolation of the organelle containing the plasma membrane from all other parts of the cell [19].

### The preparation of CCM@Ch-Cu/PtNPs

Cell membrane vesicles were mixed with 0.054 g chitosan (100–200 kDa, crab-derived) and 300 mg DSPE‑PEG2000. The mixture was subjected to extrusion using an Avanti Mini‑Extruder (with holder/heating block, Avanti catalog 610000) fitted first with a Whatman Nuclepore 0.4 μm membrane (19 mm., catalog no. 800282). Ten passes were performed between gas-tight syringes to reduce multilamellarity. Subsequently, the extruder was refitted with a 150 nm pore Track‑Etch membrane and ten additional passes performed, yielding CCM@Ch‑Cu/PtNPs of approximately 150 nm diameter. Temperature during extrusion was maintained below 80 °C via the heating block; samples were equilibrated for 5–10 min prior to extrusion. Final nanoparticle suspensions were collected in sterile vials and stored at 4 °C.

### Characterization of CCM@Ch-Cu/PtNPs

The crystallinity patterns of CCM@Ch-Cu/PtNPs using an X-ray diffractometer (XRD; Rigaku, Smart Lab, Japan). A grinder pulverized the samples into a fine powder. The 2θ was changed from 10° to 80° at a scan rate of 2°/min and a scan step of 0.1°. We used Fourier transform infrared spectroscopy (Thermo Fisher Scientific, Nicolet iS5, United States) to confirm the effective synthesis of CCM@Ch-Cu/PtNPs by analyzing different peaks. To conduct in situ XAS at the micro XAS beamline (X05LA). We acquired data for the Cu K and Pt L2 edges in fluorescence mode using silicon drift detectors. The spot size measured approximately 10 × 10 μm² [18].

### Cell culture

This research employed AGS and HGC, two human gastric carcinoma cell lines. The cell banks of the Chinese Academy of Sciences in Shanghai, China, served as the sources for both cell types. The cells were cultivated in a defined medium comprising DMEM (4.5 gm/dl glucose, sodium pyruvate, glutamax), 10% FBS (v/v), and 1% antibiotic (100 U/ml penicillin and 100 U/ml streptomycin) within a humidified atmosphere containing 5% CO_2_ at 37 °C. The media was substituted twice a day.

### Cell viability assay

Gastric carcinoma AGS and HGC cells (8 × 10³ cells/well) were seeded in 96-well plates. After 24 h, treatments were applied: CCM@Ch-Cu/PtNPs at 75 µg/mL (dispersed in sterile phosphate-buffered saline, PBS, or complete culture medium with 0.1% Tween-80 as a dispersant, sonicated for 5 min to ensure homogeneity), and doxorubicin (at defined concentration). Following a 36-h incubation, cell viability was assessed using the MTT assay, per the protocol by Periasamy et al. (2013). All treatments were conducted in triplicate with duplicates in each repeat [20].

### Colony formation assay

Post-transfection, AGS and HGC cells were maintained under normoxic or hypoxic conditions for 48 h. Cells were then harvested, and 500 cells/well were plated in six-well plates containing complete medium. Treatments were applied using the same dispersal method as in Sect. 2.6: CCM@Ch-Cu/PtNPs at 75 µg/mL in PBS or culture medium with Tween-80 and brief sonication. After two weeks’ culture, colonies were fixed with 4% paraformaldehyde and stained with crystal violet (Sigma-Aldrich, USA) before counting [21].

### DAPI & EDU staining

AGS and HGC cells were plated at 1 × 10⁵ cells per well in 48-well plates. They were treated for 24 h with CCM@Ch-Cu/PtNPs at 50 µg/mL using the same dispersion protocol (PBS or culture medium, 0.1% Tween-80, sonicated). EDU (10 µM) was added and incubated for 2 h at 37 °C. Cells were rinsed with PBS, fixed in 4% paraformaldehyde, and EDU incorporation visualized by fluorescence microscopy (Olympus CKX53). Subsequently, nuclei were stained with DAPI (1 µg/mL) at room temperature for 15 min, and fluorescence images captured.

### Cell apoptosis investigation

AGS and HGC cells were seeded in 6-well plates (3 × 10² cells/well, 200 µL medium) and incubated for 24 h. Apoptosis was evaluated using Annexin V-FITC/PI double staining. Five experimental groups were included (control; CCM alone; Cu/Pt NPs; Ch-Cu/Pt NPs; CCM@Ch-Cu/Pt NPs), each at 75 µg/mL. All nanoparticles were prepared via dispersion in PBS or complete medium with 0.1% Tween-80 and sonicated before application. After treatment, cells were washed in PBS, stained with Annexin V-FITC and PI (Millipore Corporation, Billerica, MA, USA), and analyzed by flow cytometry. Viable, early apoptotic, late apoptotic, and necrotic cell populations were quantified [22, 23].

### Intracellular ROS evaluation

Cells were treated with control, CCM, Cu/Pt NPs, Ch-Cu/Pt NPs, or CCM@Ch-Cu/Pt NPs (all at 75 µg/mL). After 0, 1, and 3 h of incubation, cells were washed once with PBS and incubated with 5 mM dihydroethidium (DHE) in serum-free medium at 37 °C for 30 min. Post-incubation, cells were rinsed with PBS and immediately imaged under a fluorescence microscope (Excitation: ~535 nm; Emission: ~610 nm) to quantify ROS-associated fluorescence intensity, following Velusamy et al. (2015) with modifications [24].

### Wound scratch assay

AGS and HGC cells were grown to ~ 85% confluence in six-well plates. A uniform scratch was made using a sterile P200 pipette tip; scratch width was standardized using a 200 µL pipette tip as a guide. After washing twice with PBS to remove debris, fresh medium containing 75 µg/mL of each nanoparticle treatment was added. Wound closure was monitored by capturing images at 0 h and 24 h (using phase-contrast microscopy), and gap width was measured using image analysis software.

### Transwell migration & invasion assays

Transwell inserts (8 μm inserts in 24-well format; Corning) were either uncoated (migration) or pre-coated with Matrigel (BD Biosciences) for invasion assays. Cells (1 × 10⁵ in 200 µL serum-free medium plus 75 µg/mL of each treatment) were seeded in the upper chambers; lower chambers contained medium supplemented with 15% FBS as chemoattractant. After 24 h incubation at 37 °C, non-migrated cells were removed, and the membranes fixed with 4% formaldehyde for 15 min, stained with 1% gentian violet for 20 min, rinsed, and imaged. Migrated/invaded cells were counted in five random fields per membrane [25].

### qRT-PCR analysis

The total RNA was extracted from the cells and tissues using Trizol reagent (Takara, Japan). Takara’s (Dalian, China) Reverse Transcriptase Kit was used to convert RNA into cDNA. ABI 7300 equipment (Applied Biosystems, Foster, CA) was used to perform quantitative reverse transcription polymerase chain reaction (qRT-PCR) with Takara’s SYBR Premix Ex Taq. 2 − Ct approach was used to measure the relative expression of mRNAs, utilizing GAPDH as an internal reference [26].

### ATP quantification

Cells (AGS, HGC) were seeded in white, opaque 96-well plates (1-2 × 10^4^ cells/well), allowed to adhere overnight, and treated for 24 h as in JC-1 assays. ATP was measured using a luminescent assay (e.g., CellTiter-Glo^®^; Promega) according to the manufacturer’s protocol. Luminescence was read on a microplate reader, background-subtracted, and normalized to protein content (BCA from matched wells) or cell number (crystal violet nuclei count). Data are presented as % of vehicle control.

### Cytochrome-c release

After 24 h treatment, cells were harvested on ice and fractionated into cytosolic and mitochondrial fractions using a digitonin-based kit or differential centrifugation (10 min at 700 g to remove nuclei/debris; 10 min at 10,000 g to pellet mitochondria). Cyt-c levels were assessed by Western blot (anti-cytochrome-c; cytosolic marker GAPDH; mitochondrial marker COX IV) or by sandwich ELISA on cleared cytosols. Cyt-c release is reported as (cytosolic Cyt-c / total Cyt-c) or as cytosolic Cyt-c normalized to GAPDH.

### Xenograft gastric cancer mouse model and treatment protocol

All animal experiments were approved by in accordance with ARRIVE guidelines for the use of experimental animals. Animal Ethics Committee of Shanxi Provincial Cardiovascular Hospital, NO.20252YY108. Male C57BL/6 mice (21-25 g) were randomly divided into five groups (n = X per group): Control, DSS + Vehicle, CCM, Cu/PtNPs, and CCM@Ch-Cu/PtNPs. Gastric cancer was established using a xenograft model. Briefly, AGS and HGC human gastric cancer cells (1 × 10⁶ cells suspended in 100 µL PBS) were subcutaneously injected into the right flank region of mice. Tumor formation was monitored weekly, and tumor volume was measured using digital calipers and calculated using the formula:

$${\rm{Tumor\,volume}} = {1 \over 2}\,\,{\rm{length}} \times {\rm{widt}}{{\rm{h}}^2}$$

The treatment started when the tumors were about 80-100 mm³ in size. To replicate a tumor microenvironment linked to inflammation, mice in the designated groups received 2.5% dextran sulfate sodium (DSS) in their drinking water for 7 consecutive days prior to therapeutic intervention. DSS therapy was utilized to induce systemic inflammatory stress rather than to facilitate gastric carcinogenesis. After that, the mice were given nanoparticles (100 mg/kg) or a vehicle control through an IV for ten days in a row. Body weight and tumor growth were tracked during the course of treatment. On the 11th day after treatment started, blood samples were taken using retro-orbital puncture and centrifuged (4 °C, 3000 rpm, 15 min) to separate the serum. Resected, weighed, and histopathologically evaluated were tumor tissues, colon, and spleen [27]. 

### Evaluation of histopathology

Mice were euthanized between days 145 and 173 of the experiment. The esophagus and stomach of the mice were excised as a monobloc via a thoracoabdominal incision. Each animal’s stomach was incised along the larger curvature, affixed to a cork plate, and preserved in 10% formaldehyde for 48 h. The stomach was subsequently separated and sectioned into four or five transverse slices along the longitudinal axis. The sections were conditioned in two or three plastic capsules for processing and embedded in paraffin to encompass the complete extension of the organ in the preparation. Sections of the stomach were stained with hematoxylin-eosin and examined for normal histology and adenocarcinoma (ACA).

### Statistical analysis

All quantitative data are presented as the average, accompanied by the standard deviation (mean ± SD). All experiments were conducted in replicates. Comparisons among multiple groups were conducted using one-way analysis of variance (ANOVA) followed by Tukey’s post-hoc test to determine pairwise differences. Notable distinctions between the groups were denoted by statistical significance *P* values are reported, * (*P* < 0.01), *** (*P* < 0.001), or **** (*P* < 0.0001).

## Results and discussion

### Characterization of CCM@Ch-Cu/PtNPs

We successfully synthesized Cu/Pt bimetallic nanoparticles with a 1:1 atomic ratio using a solvothermal method. The process utilized high-purity precursors in combination with benzyl ether as the solvent. These nanoparticles, due to their precise size and composition, are well-suited for applications in catalysis, imaging, drug delivery, and biomimetic platforms for cancer treatment. Coordination bonding, electrostatic stabilization, and polymer-assisted nucleation are the main ways that chitosan-coated Cu/Pt nanoparticles come together. Chitosan is a cationic polysaccharide made up of β-(1→4)-linked D-glucosamine units with a lot of amino (-NH₂) and hydroxyl (-OH) groups. These functional groups serve as metal-chelating sites by coordinating with Cu²⁺ and Pt²⁺/Pt⁴⁺ ions through lone-pair electron donation, establishing stable metal-ligand complexes before reduction. When the coordinated metal ions are chemically reduced, they nucleate and proliferate while still being held in place by the chitosan matrix. This makes Cu/Pt bimetallic nanoparticles that are evenly spread out. The amino groups control the size of nanoparticles by keeping them in a small space and keeping them from clumping together by using steric hindrance and electrostatic repulsion from protonated -NH₃⁺ groups. Chitosan serves as both a reducing-assisting stabilizer and a soft template that shapes the structure of nanoparticles [28]. In bimetallic systems, Cu and Pt can create alloyed or surface-segregated domains based on reduction kinetics. This speeds up the passage of electrons across metals and makes it easier for ROS to be generated. Moreover, the positive surface charge of chitosan facilitates cellular uptake through electrostatic interactions with negatively charged cancer cell membranes, hence enhancing therapeutic efficacy. Chitosan serves a dual purpose in the structural stabilization and functional enhancement of Cu/Pt catalytic nanotherapeutics. The produced Cu/Pt nanoparticles predominantly exhibit sizes ranging from 7 to 10 nm, as ascertained using TEM histogram analysis. Following the stabilization of chitosan and the application of cancer cell membrane coating (CCM@Ch-Cu/PtNPs), the overall hydrodynamic diameter increased, as anticipated due to the stacking of the polymer and membrane, while preserving nanoscale dispersion and uniform morphology.

**Fig. 1:**
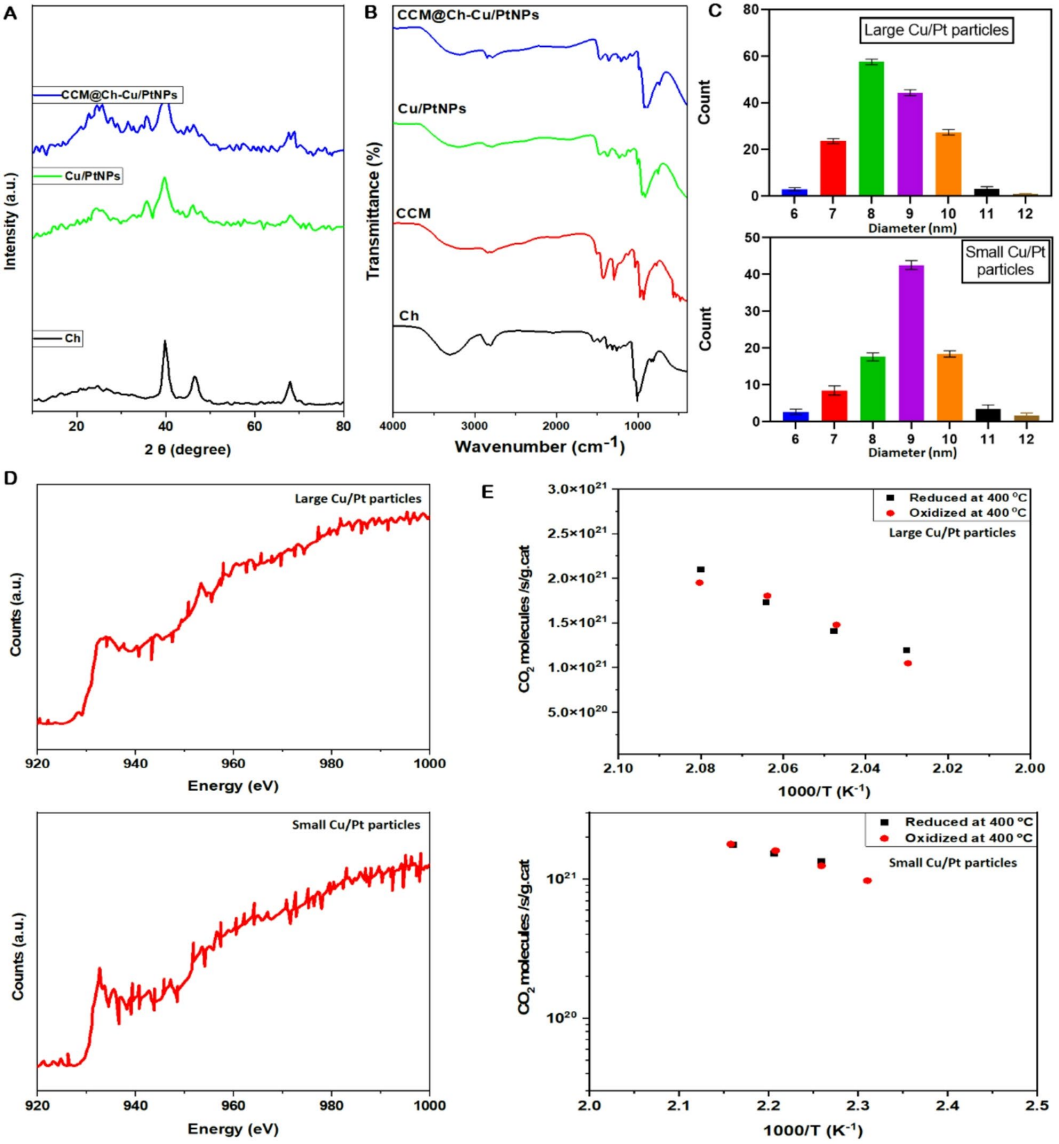
(**A**) X-ray diffraction (XRD) patterns confirming the crystalline nature of synthesized nanoparticles: pure Chitosan (Ch), Cu/PtNPs, and CCM@Ch-Cu/PtNPs composites. The shift and broadening of peaks in CCM@Ch-Cu/PtNPs indicate successful nanoparticle embedding and compositional change; (**B**) FTIR spectrum shows the peak of tested samples; (**C**) Histograms showing size distribution of large and small Cu/Pt particles analyzed via TEM, highlighting predominant diameters between 7–10 nm, indicating polydispersity; (**D**) X-ray absorption near edge structure (XANES) spectra depicting the electronic state and local structure of Cu/PtNPs; (**E**) Arrhenius plots showing CO oxidation activity of Cu/PtNPs under reduced (red) and oxidized (blue) conditions, reflecting excellent catalytic stability and thermal durability

Further these nanomaterials are confirmed by following characterization techniques. The XRD pattern of CCM@Ch-Cu/PtNPs showed 2θ = 21.68⁰, 30.94⁰, 45.33⁰,53.95⁰ and 63.94⁰ confirming the presence of Cu/Pt NPs in the formulated nanocomposites. The X-ray diffraction (XRD) patterns validate the successful synthesis of CCM@Ch-Cu/PtNPs. The crossing and aggregated spectrum characteristics of CCM@Ch-Cu/PtNPs affirm the existence of both chitosan and cancer cell membrane constituents on the nanoparticle surface, hence substantiating the successful construction of the biomimetic nanoplatform. The CM@Ch-Cu/PtNPs spectra integrate the characteristic peaks of chitosan and CCM, validating the effective coating of the bimetallic nanoparticles with both the polymer and the cell membrane. The retention of essential functional group peaks (amide, hydroxyl, and saccharide bands) signifies the structural integrity of the coating materials during nanoparticle assembly. The distinct diffraction peaks are indicative of the crystalline phases of Cu and Pt nanoparticles. The incorporation of the chitosan (Ch) coating and cancer cell membrane (CCM) does not substantially modify the crystal structure, signifying that the core-shell architecture is preserved. The particle surface analysis elucidates the size distribution of Cu/Pt nanoparticles. The “large Cu/Pt particles” primarily range in size from 8 to 10 nm, whereas the “small Cu/Pt particles” exhibit a more restricted size range of 6 to 8 nm. This illustrates consistency in particle size for both large and small particles, which is crucial for reproducibility in biomedical applications. The histogram illustrates the uniformity in size distribution. It highlights the limited size dispersion, signifying well-regulated synthesis conditions. The plasma membrane has a phospholipid bilayer around 7-10 nm in thickness, and passive diffusion is often limited to very small molecules (< 1 nm). Nanoparticles measuring 7-100 nm do not penetrate the membrane through pores; rather, they are ingested through active cellular absorption mechanisms. This consistency is essential for attaining dependable therapeutic and diagnostic efficacy. Stability assessments were performed at two temperatures: room temperature (RT) and elevated temperature (40 °C). The findings indicate that Cu/Pt nanoparticles exhibit stability under these conditions, as evidenced by the uniform CO adsorption values. This stability guarantees the nanoparticles’ effectiveness during storage and application were shown in Fig. 1. Similar to this result suggests that the platinum crystal lattice in Cu@Pt/C 0.5 is expanding. Additionally, bioactive compounds in the extract acted as both stabilizing and reducing agents for platinum ions. Consequently, it validated the efficient green manufacturing of Pt nanoparticles. The average particle size and PDI of the environmentally synthesized Pt nanoparticles were determined to be 102.2 nm and 0.187, respectively. It demonstrates pronounced peaks at 2θ = 12.45°, 16.97°, and 64.09°, confirming the synthesis of crystalline Pt-NPs via the green technique [29]. XRD results showed that the synthesized electrocatalysts had a core that was high in copper and a shell that was high in platinum. The XRD tests revealed the absence of diffraction peaks associated with the core metal and the movement of several diffraction planes towards higher 2q values than pure metals [10]. The X-ray Absorption Spectroscopy (XAS) data for Cu exhibit distinct absorption characteristics during the processes of reduction, oxidation, and re-reduction at 800 °C. The alterations in energy indicate variations in the oxidation states of Cu, validating its redox versatility within the Cu/Pt nanoparticles. The Pt XAS spectra demonstrate stable electronic transitions, underscoring the durability of Pt during redox cycling. The peaks indicate the retention of Pt’s catalytic activity despite numerous redox cycles. The XAS profile of chitosan exhibits negligible variations, signifying its structural integrity under the experimental conditions. This validates its efficacy as a stabilizing and coating agent for nanoparticles. The CCM@Ch-Cu/PtNPs had a sustained and greater drug release profile than the others. CCM@Ch-Cu/PtNPs released 90% of their medication at 72 h, possibly due to the biomimetic cancer cell membrane and chitosan. Uncoated Cu/PtNPs released 60% of the loaded drug over the same period, demonstrating that chitosan modification moderately enhances drug release control. The Ch (chitosan-only) group showed low drug release (< 20%), indicating limited drug loading or diffusion without the Cu/Pt core. These findings show that CCM@Ch-Cu/PtNPs enable sustained release and increase drug availability, which may improve therapeutic efficacy in vivo by prolonging and targeting tumor site drug exposure Sup. Figure 1.

**Fig. 2:**
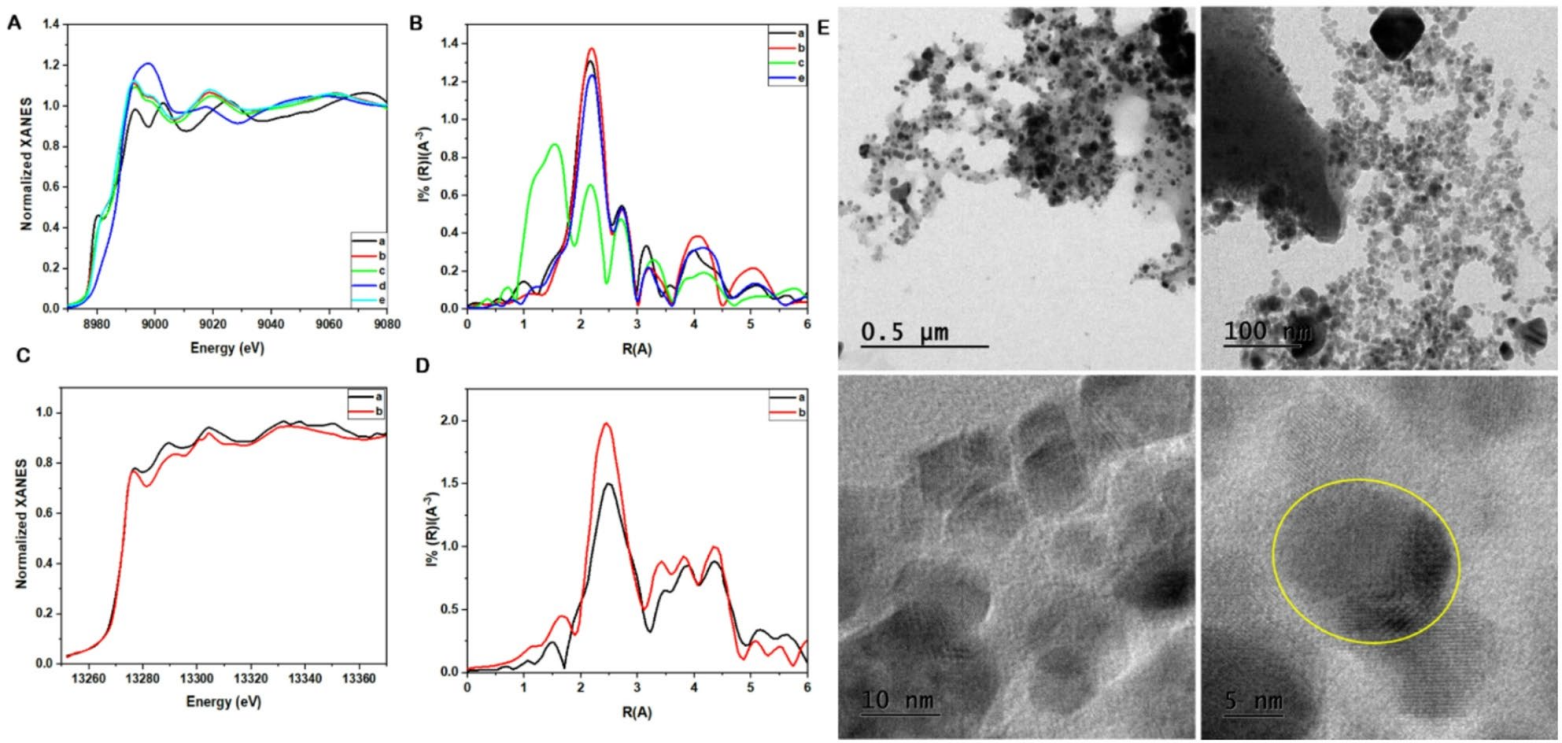
XAS and TEM Analysis of Cu/Pt Nanoparticles and CCM@Ch-Cu/PtNPs. (**A**-**D**) XAS data showing the reduction, oxidation, and re-reduction processes at 800 °C for (**a**) Cu, (**b**) Pt, (**c**) Ch, (**d**) Cu/Pt NPs, and (**e**) Ch-Cu/PtNPs. (**E**) TEM images of CCM@Ch-Cu/PtNPs at various magnifications, highlighting the nanoparticle morphology

The integrated Cu/Pt XAS data demonstrate synergistic interactions between Cu and Pt. The unique peaks confirm the successful synthesis of bimetallic nanoparticles, indicating improved stability and potential catalytic capabilities under extreme conditions. The TEM images confirm the nanoscale uniformity and core-shell morphology of CCM@Ch-Cu/PtNPs, which are crucial for improved functionality in biomimetic applications. The integration of XAS and TEM findings substantiates the viability of CCM@Ch-Cu/PtNPs as a stable and efficient nanoplatform for targeted cancer therapy, exhibiting improved biocompatibility and catalytic characteristics attributed to the chitosan and cancer cell membrane coatings (Fig. 2).

**Fig. 3:**
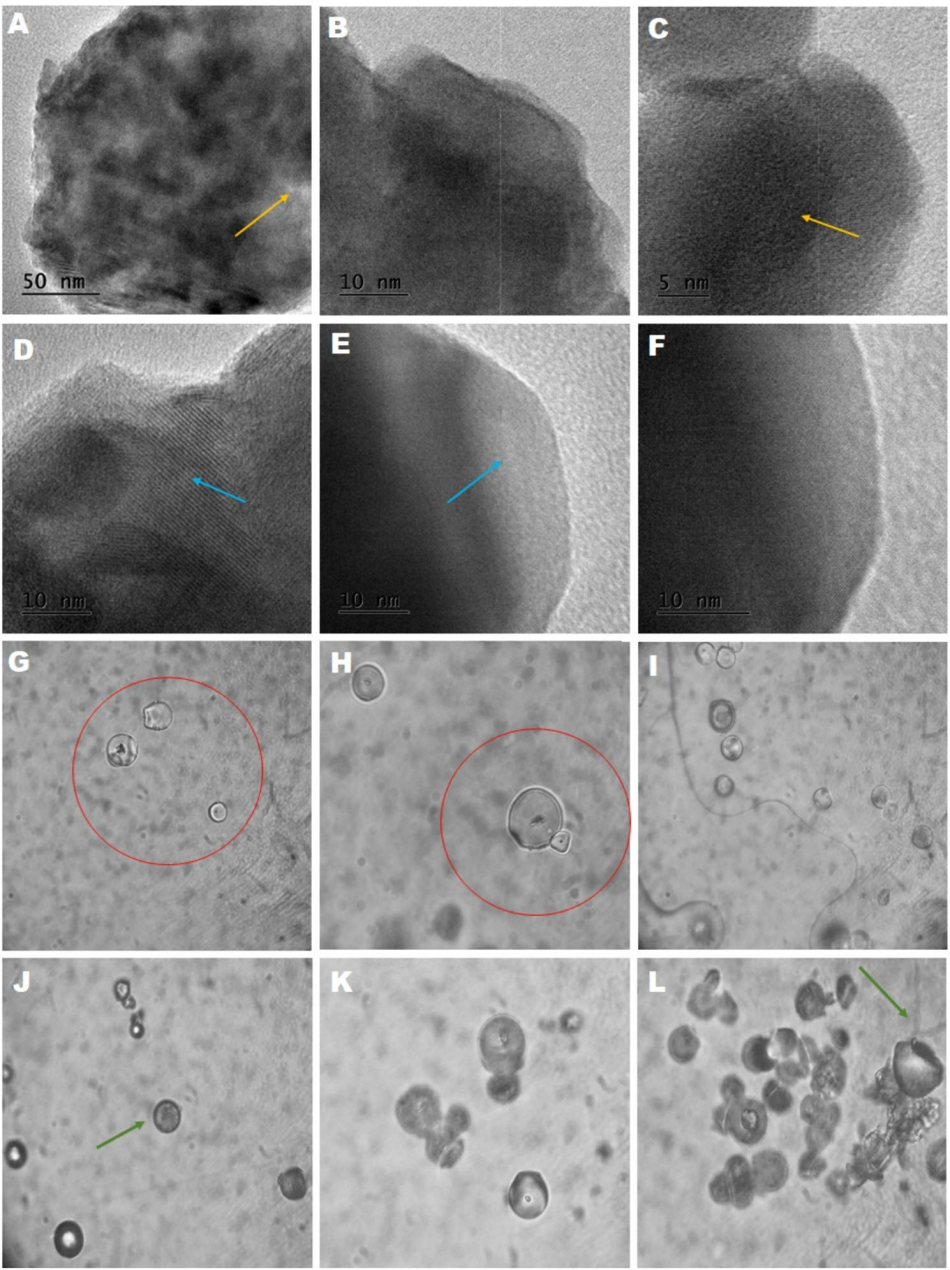
Transmission electron microscopy analysis of CCM@Ch-Cu/PtNPs nanoparticle morphology, membrane interaction, and cellular internalization. (**A**-**C**) TEM images showing the morphology of Cu/Pt nanoparticles, where yellow arrows indicate dense metallic nanoparticle cores; (**D**-**F**) High-resolution TEM images demonstrating the core–shell architecture of CCM@Ch-Cu/PtNPs, with blue arrows highlighting the peripheral coating layer corresponding to chitosan and cancer cell membrane components; (**G**-**I**) TEM images showing nanoparticles attached to the plasma membrane of gastric cancer cells (red circles), indicating nanoparticle–membrane interaction; (**J**-**L**) Intracellular vesicles containing nanoparticles (green arrows), demonstrating endocytosis-mediated internalization of CCM@Ch-Cu/PtNPs. Scale bars 50 nm, 10 nm, and 5 nm

When it comes to diagnosing diseases, monitoring conditions, evaluating prognoses, and evaluating efficacy, biomarkers derived from serum had many physiologically active components especially proteins and metabolites can provide valuable insights into the body’s health status and the evolution of sickness. Hyperproliferation and invasive metastases of the gastric mucosa characterize gastric cancer (GC), a neoplastic disorder of the stomach [30]. Likewise, the alteration of CCM resulted in a substantial reduction in CCM dispersion and a marked increase in DDS accumulation within the tumor tissue [31]. In contrast to the control group, administration of 40 and 60 µM of oleanolic acid (OA) significantly increased the proportion of early and late apoptosis. The PARP protein, which is involved in DNA repair, had fragmented and there was a noticeable increase in the expression of Bax, a protein that promotes cell death [15]. The HeLa and HepG2 cells show dose-dependent viability effects when exposed to Pt-NPs and their DOX conjugates. The cell viability was significantly higher than 80%, and none of the NPs had any negative effects on the three cell lines. This indicates that the Pt-NPs stabilized with plant extract are safe for human consumption and do not cause any harm [6]. The copper and Pt electrocatalysts exhibited localized aggregation in certain regions of platinum. When combined with copper, the diminutive nanoparticles coalesced in specific regions which leads to the formation of loose aggregate. It is challenging to tell carbon support from metal nanoparticles since the former can add color to the latter without showing a clearly defined spherical form. Cu@Pt/C electrocatalysts exhibited a weakening of multiple Pt diffraction peaks in XRD patterns as the copper content increased [32]. Through the application of severe temperatures to both small and large Cu/Pt particles, the current study explored the process of converting CO to CO_2_ offering an enhanced comprehension of the dynamics inherent in the oxidation-reduction-reoxidation cycle for catalytic CO conversion. This study provides critical insights into the influence of particle size and thermal treatments on enhancing catalytic efficacy for CO oxidation. Following the oxidation (black) and reduction (red) processes, we noted the presence of both big and small particles. Next, the re-reduction of copper particles (pink) was investigated (Sup Fig. 2). Transmission electron microscopy analysis of CCM@Ch-Cu/PtNPs nanoparticle morphology, membrane interaction, and cellular internalization. (A-C) TEM images showing the morphology of Cu/Pt nanoparticles, where yellow arrows indicate dense metallic nanoparticle cores; (D-F) High-resolution TEM images demonstrating the core–shell architecture of CCM@Ch-Cu/PtNPs, with blue arrows highlighting the peripheral coating layer corresponding to chitosan and cancer cell membrane components; (G-I) TEM images showing nanoparticles attached to the plasma membrane of gastric cancer cells (red circles), indicating nanoparticle–membrane interaction; (J-L) Intracellular vesicles containing nanoparticles (green arrows), demonstrating endocytosis-mediated internalization of CCM@Ch-Cu/PtNPs. Scale bars 50 nm, 10 nm, and 5 nm (Fig. 3).

### In vitro analysis of CCM@Ch-Cu/PtNPs

**Fig. 4:**
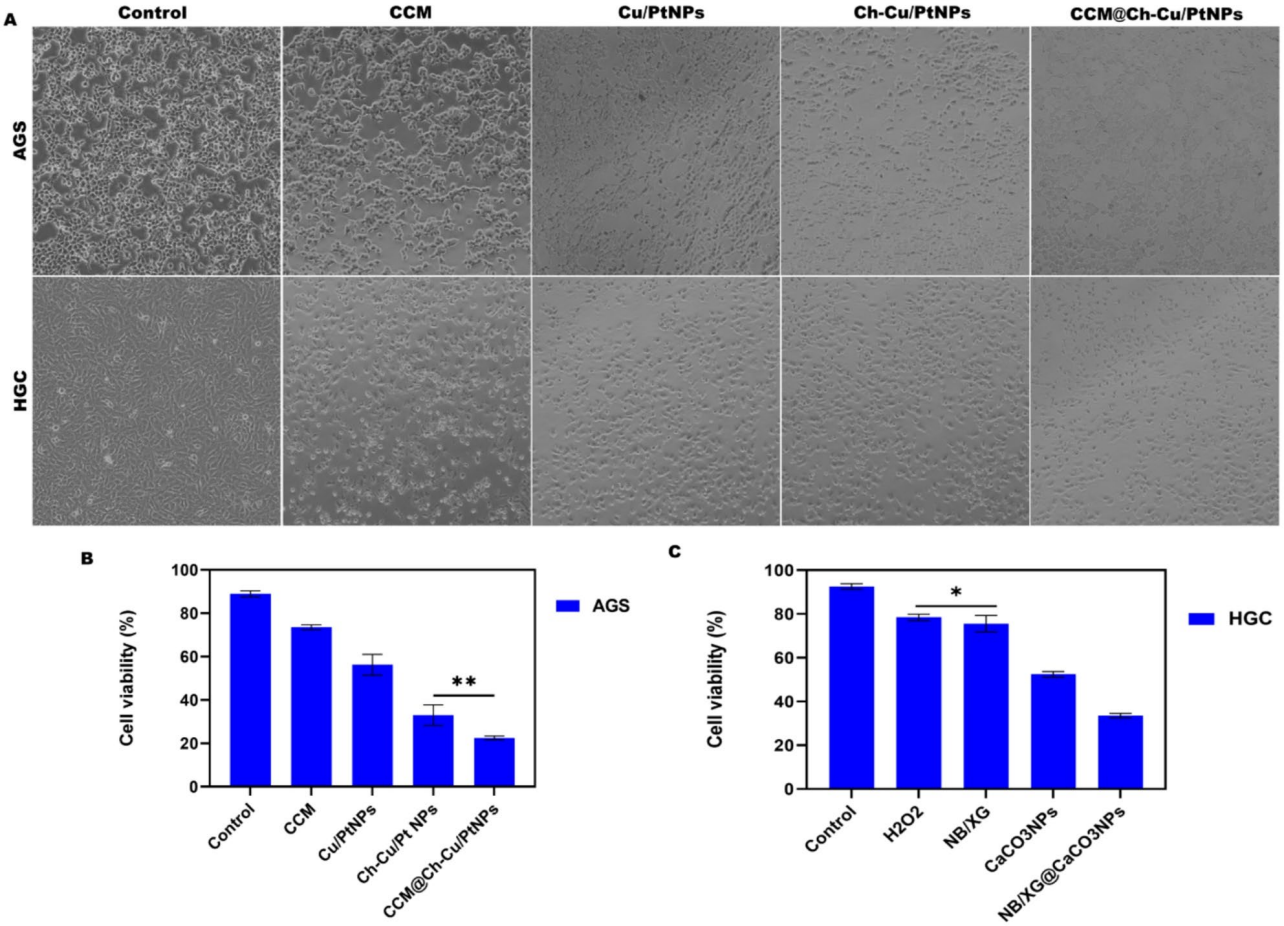
Cell Viability Assay in AGS and HGC Cell Lines. (**A**) Representative cell viability data for AGS and HGC cells treated with CCM, Cu/PtNPs, Ch-Cu/PtNPs, and CCM@Ch-Cu/PtNPs. (**B**-**C**) Statistical analysis of cell viability as a percentage of control. Values are expressed as mean ± SD. Asterisks indicate statistical significance, with *p < 0.01 and **p < 0.001

A. Representative phase-contrast microscopy images showing the morphological response of AGS (top row) and HGC (bottom row) gastric cancer cells after treatment with: Control (untreated), CCM alone, Cu/Pt nanoparticles, Ch-Cu/Pt nanoparticles, and CCM@Ch-Cu/Pt nanoparticles (all at 75 µg/mL). Images were captured after a 24-hour incubation, illustrating progressive loss of viable cells and morphological alterations with increasing complexity of nanoparticle treatment. (B) Quantitative analysis of AGS cell viability expressed as a percentage relative to control. Bars represent the mean ± SD from three independent experiments, each performed in duplicate. Treatment with Ch-Cu/Pt NPs significantly reduced viability (p < 0.001), and CCM@Ch-Cu/Pt NPs led to the most pronounced decline (p < 0.001). (C) Quantitative analysis of HGC cell viability (mean ± SD). CCM@Ch-Cu/Pt NPs again significantly decreased viability, indicated by p < 0.01 compared to other treatments (Fig. 4). Similarly, different doses significantly reduced the viability of the gastric cancer cell line (AGS). Results showed that cell proliferation significantly decreased once gossypol treatment began, and this effect was dose- and time-dependent [33]. ROS generation is visually represented with increased fluorescence intensity in both AGS and HGC cell lines. The control group showed minimal fluorescence, indicating negligible ROS levels. Treatment with CCM induced a slight increase in ROS generation. Cu/PtNPs showed moderately increased fluorescence intensity, suggesting their role in enhancing ROS. Ch-Cu/PtNPs further elevated ROS levels, likely due to enhanced stability and interaction with cells mediated by chitosan. The CCM@Ch-Cu/PtNPs group exhibited the highest fluorescence intensity, signifying the most significant ROS generation.

**Fig. 5:**
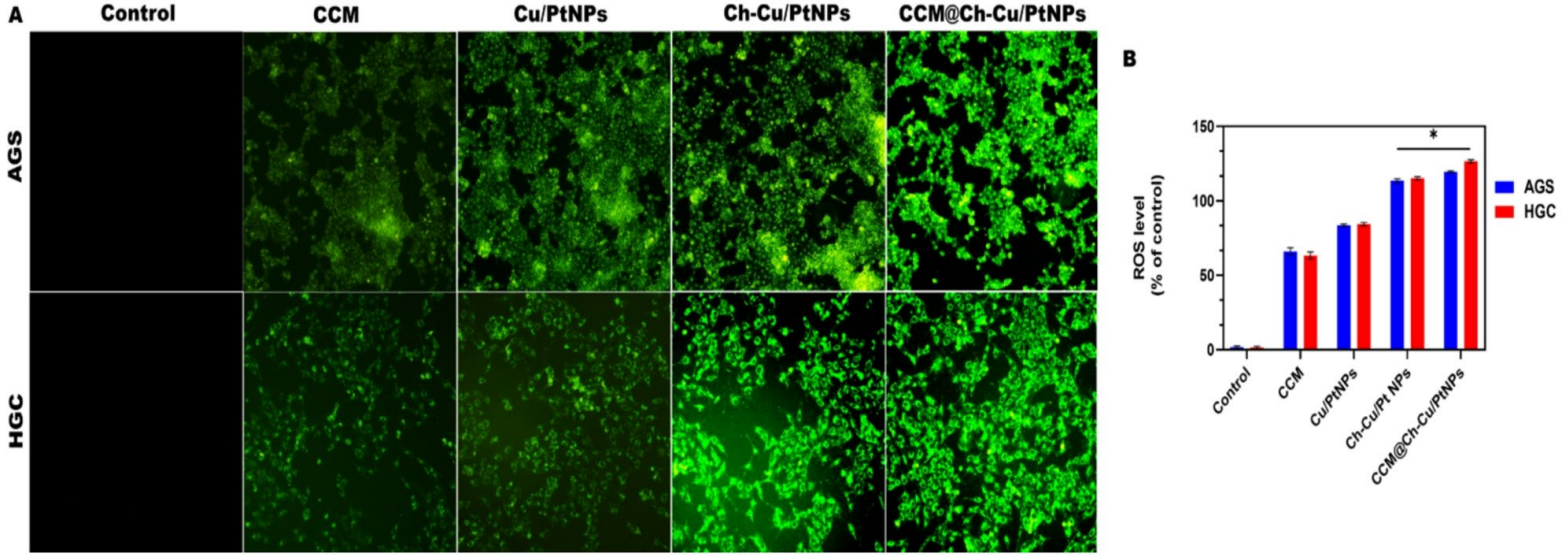
ROS Activity in AGS and HGC Cell Lines. (**A**) Assessment of ROS generation in AGS and HGC cells treated with CCM, Cu/PtNPs, Ch-Cu/PtNPs, and CCM@Ch-Cu/PtNPs. (**B**) Statistical analysis of ROS levels as a percentage of control. Values are presented as mean ± SD. Asterisks indicate statistical significance (*p < 0.01)

A. Representative fluorescence microscopy images illustrating ROS generation in AGS (top row) and HGC (bottom row) cells following treatment with CCM alone, Cu/Pt NPs, Ch-Cu/Pt NPs, and CCM@Ch-Cu/Pt NPs (all at 75 µg/mL). Enhanced green fluorescence corresponds to elevated ROS levels in treated cells. (B) Quantitative analysis of ROS levels expressed as a percentage relative to control. Bars represent mean ± SD from at least three independent experiments. The CCM@Ch-Cu/Pt NPs induced a statistically significant increase in ROS production (p < 0.01), surpassing that observed with other nanoparticle formulations. The elevated ROS levels are attributed to the synergistic effects of the cancer-cell-membrane coating and chitosan modification. The biomimetic CCM layer promotes tumor-cell homotypic targeting and efficient uptake (Fig. 5). Similarly, elevated stages of ROS elicited by the therapeutic agents 5-fluorouracil and oxaliplatin are responsible for their viability [34]. According to similar outcome reports, the tumor microenvironment (TME) is slightly acidic, making it easier for 5-FU and chitosan to move around and kill many SMMC-7721 cells. Interestingly, when exposed to near-infrared light, cell viability drops much more [35]. One common In vitro experiment that uses a cell’s ability to form a colony to evaluate a drug’s potential for cell reproduction is the clonogenic assay. This test determines how many uncontrolled cell divisions in each cell population are capable of undergoing pharmaceutical applications [12]. In SGC-7901 cells, shikonin therapy reduced expression levels of PCNA and cyclin D1, suggesting that shikonin can limit cell proliferation and colony formation via miR-96, whereas overexpressing miR-96 increased cell proliferation and colony formation [36].

**Fig. 6:**
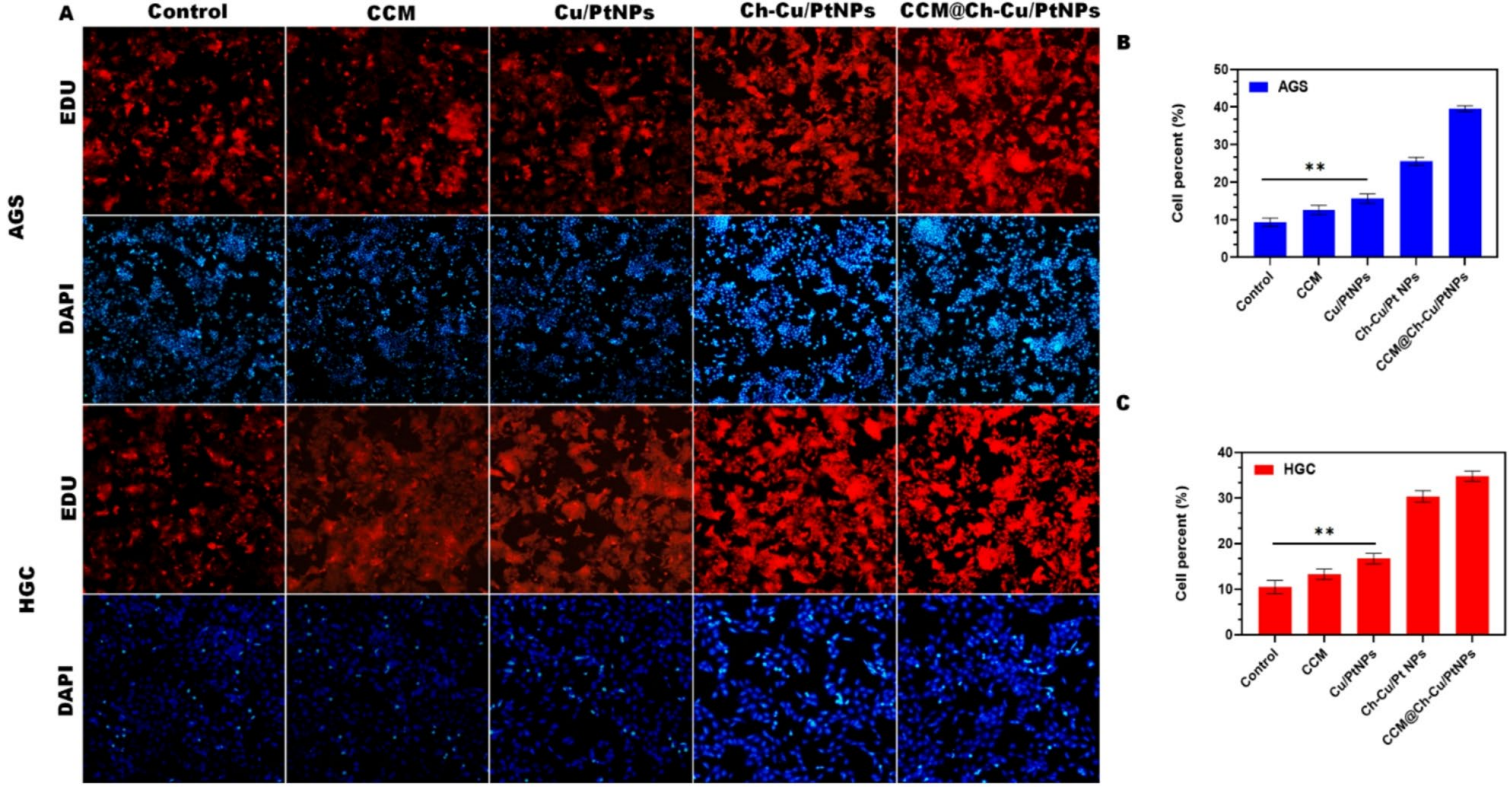
Effect of CCM, Cu/PtNPs, Ch-Cu/PtNPs and CCM@Ch-Cu/PtNPs on the apoptosis of AGS and HGC cells. Cells were treated with different samples for 24 h. After treatment, (**A**) EdU incorporation assay was used to assess cell apoptotic ability (**A**); The percent of cells are calculated as significantly analyzed. Asterix **showed statistical significance at *p* < 0.01

**Fig. 7:**
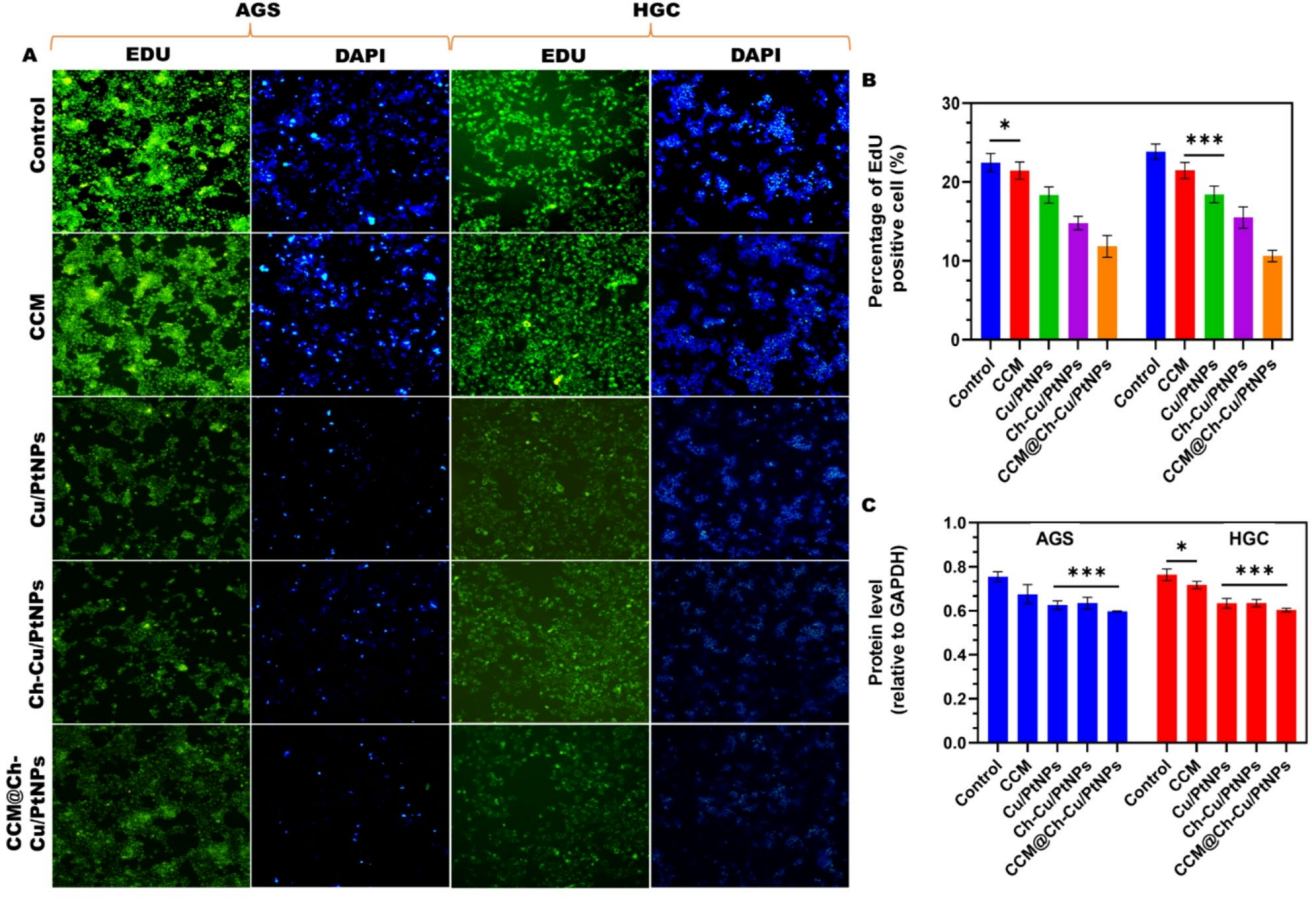
Effect of CCM, Cu/PtNPs, Ch-Cu/PtNPs and CCM@Ch-Cu/PtNPs on AGS and HGC cell lines with EDU and DAPI incorporated assays for 24 h (**A**); The percentage of EDU positive cells and protein level of expression has been showed (**B**-**C**). Data are expressed as mean ± standard error of the mean. Asterix *** has *p* < 0.0001, ** showed *p* < 0.001 and *p* < 0.01

**Fig. 8:**
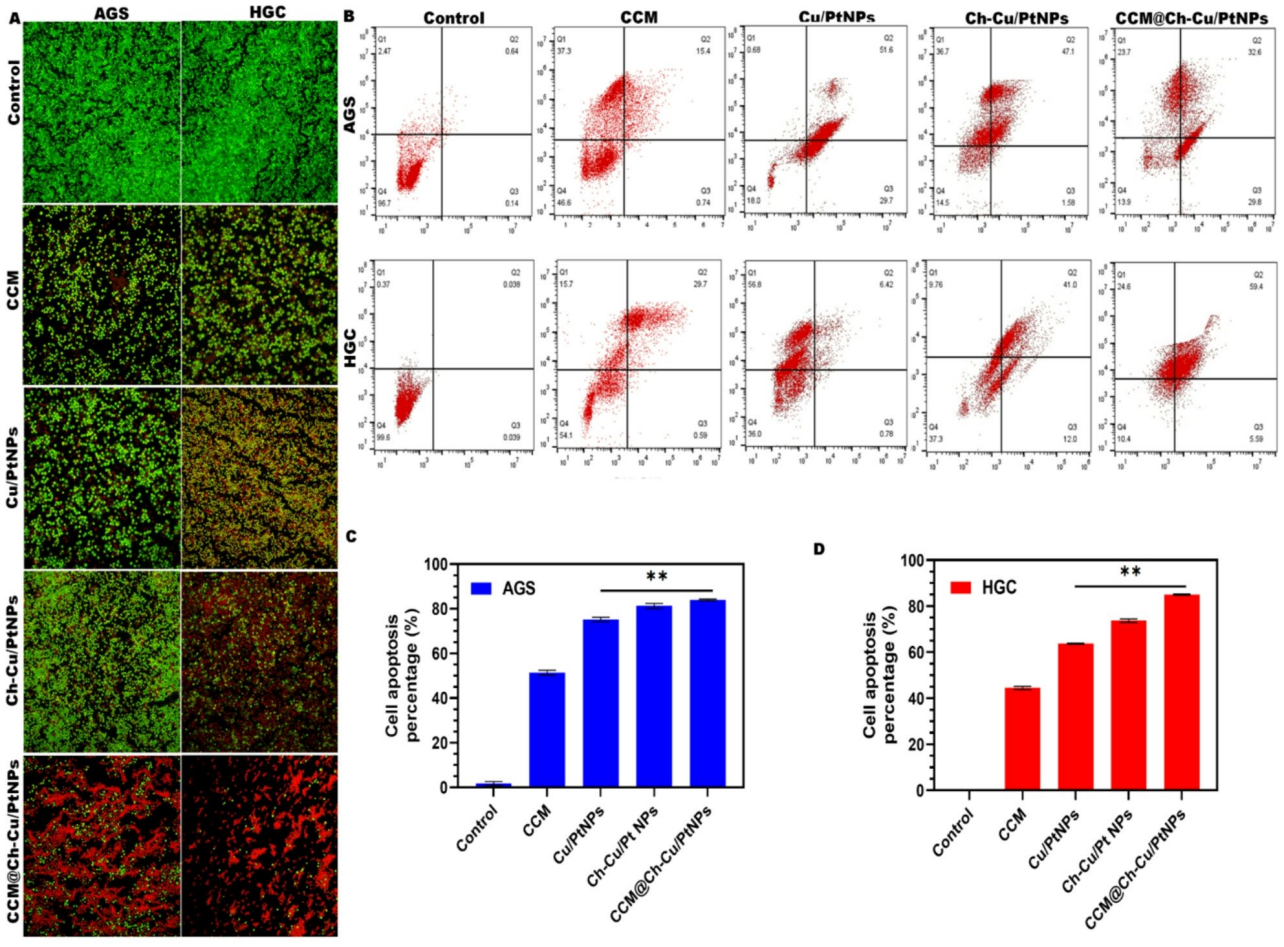
Live and dead cell percentage of AGS and HGC cell lines treated with CCM, Cu/PtNPs, Ch-Cu/PtNPs and CCM@Ch-Cu/PtNPs (**A**); Apoptosis rate on AGS and HGC treated with different samples (**B**); Apoptotic percentage of AGS and HGC has showed statistically significance among group *p*<0.0.001

**Fig. 9:**
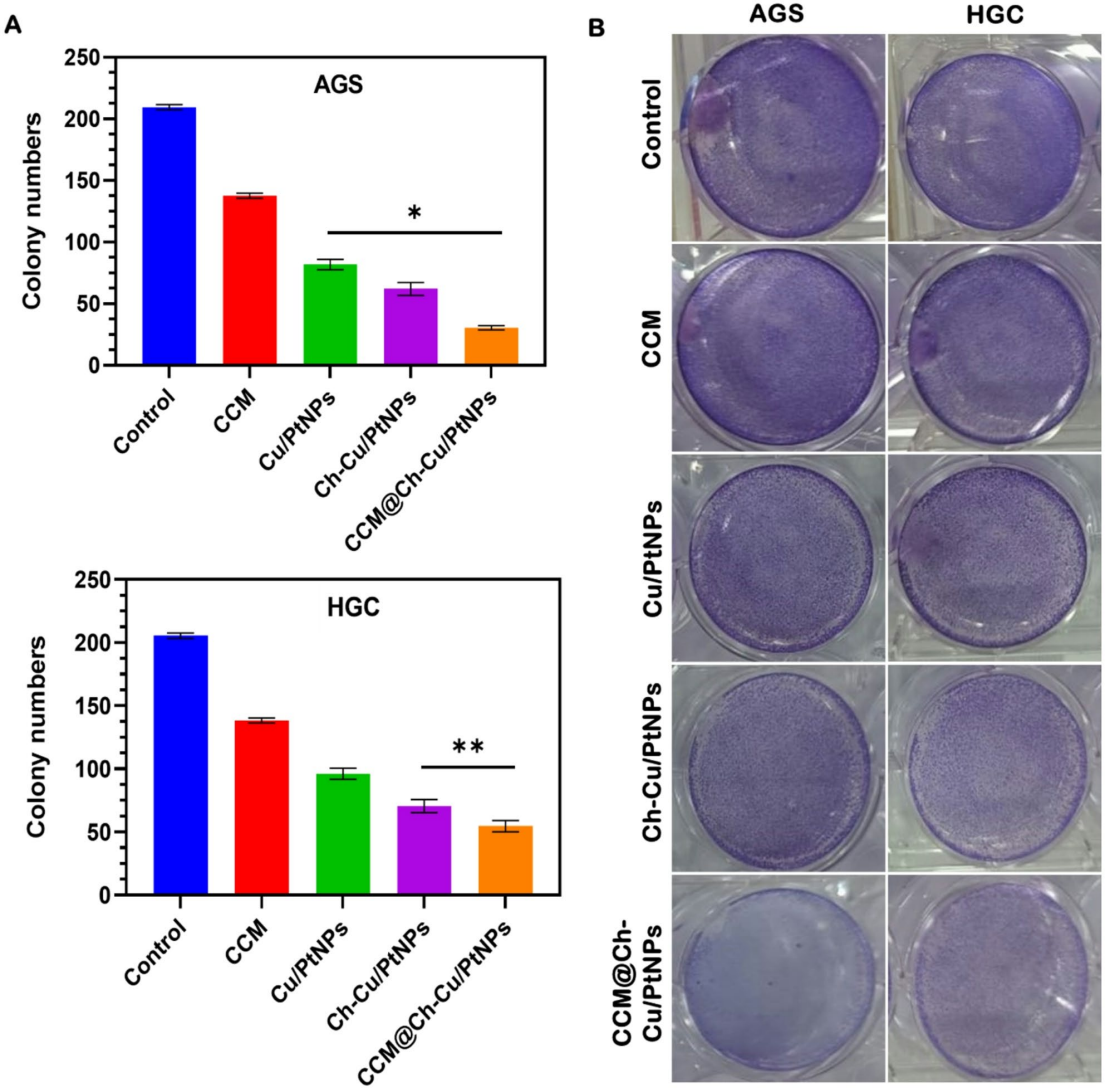
Colony number of AGS and HGC (**A**); Colony formation unit treated with CCM, Cu/PtNPs, Ch-Cu/PtNPs and CCM@Ch-Cu/PtNPs (C)

The effect of CCM, Cu/PtNPs, Ch-Cu/PtNPs and CCM@Ch-Cu/PtNPs on the apoptosis of AGS and HGC cells. Cells were treated with different samples for 24 h. After treatment, (A) EdU incorporation assay was used to assess cell apoptotic ability (A). The percent of cells are calculated as significantly analyzed (Fig. 6). We examined the impact of CCM, Cu/PtNPs, Ch-Cu/PtNPs, and CCM@Ch-Cu/PtNPs on the apoptosis of AGS and HGC cells and treated the cells with different samples for 24 h. We evaluated cell apoptotic capabilities after treatment using an EdU incorporation test. Moreover, we examined the impact of CCM, Cu/PtNPs, Ch-Cu/PtNPs, and CCM@Ch-Cu/PtNPs on AGS and HGC cell lines using EDU and DAPI incorporated assays for 24 h. for 24 h. Figure 7 displays the percentage of EDU-positive cells and the expression level of proteins. AGS and HGC cell lines treated with CCM, Cu/PtNPs, Ch-Cu/PtNPs, and CCM@Ch-Cu/PtNPs show the percentages of live and dead cells. The rate of highest apoptosis in AGS and HGC treated with various samples is examined. The apoptotic percentage of AGS and HGC has shown statistically significant differences among groups (Fig. 8). Similarly, xanthohumol (Xn) treatment may induce apoptosis and inhibit growth, resulting in a decrease in the total cell count. The AGS cells’ ability to metastasize in the context of Xn therapy showed that Xn slowed down wound healing, cell migration, and invasion in a way that depended on the dose, which suggests that Xn stops AGS cells from metastasizing. Transwell test counted the invading and migrating cells. The amount of migrating and invading cells was significantly and dose-dependently decreased after 24 h of Xn treatment [25]. The bar graphs demonstrate a significant reduction in the number of colonies of AGS and HGC cells formed upon treatment. The control group (blue) exhibited the highest colony count, followed by CCM, Cu/PtNPs, Ch-Cu/PtNPs, and CCM@Ch-Cu/PtNPs, demonstrating the most substantial inhibitory effect. The corresponding images visually confirm the quantitative data. The control group shows densely stained colonies, while the CCM@Ch-Cu/PtNPs group exhibits almost no staining, indicating the inhibition of colony growth (Fig. 9). The abundance of CASC9 in gastric cancer tissue was approximately eight times greater than in other tissues, and the increased expression of CASC9 correlated with extensive invasion. Furthermore, Wu et al. discovered that the upregulation of CASC9 enhances esophageal squamous cell carcinoma via negatively modulating expression [37]. In line with the results, miR-370 expression in tissues from stomach cancer was lower in 46 individuals who had gastric resection [38]. miR-370 mimics substantially reduced the protein levels, while this inhibitor dramatically elevated these protein levels [39]. The DAPI staining of cell nuclei showed that core-triple shell-treated cells had more DNA damage than drug-treated cells treated only with one drug [40]. According to flow cytometry data, treatment with core-shell nanoparticles significantly increased cell apoptosis and necrosis. In particular, late apoptosis showed the most significant increase (38.5-fold), suggesting that apoptosis induction is the nanoparticles’ principal anticancer strategy in gastric cancer cells [41]. The effects of Tanshinone IIA (Tan-IIA) on the protein expression levels of EGFR, AKT, mTOR, p-TEN, and β-actin were evaluated in tumors derived from AGS cells. Protein expression was analyzed after administering varying doses of Tan-IIA to AGS cell xenograft tumors [42].

**Fig. 10:**
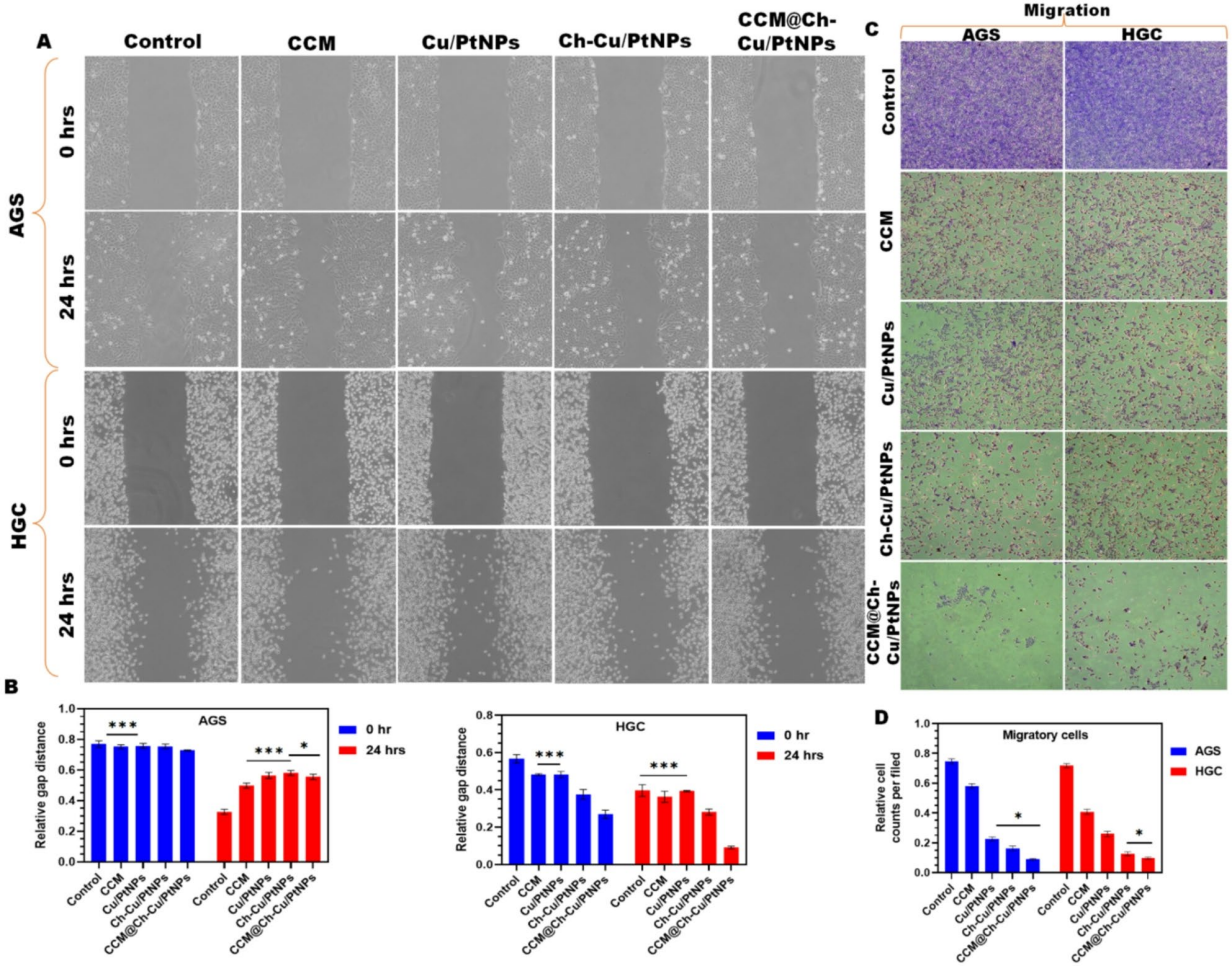
The effect of CCM@Ch-Cu/PtNPs on wound scratch assay on AGS and HGC cells treated with different concentrations for 6 and 12 h, followed by measurement of the relative wound width (**A**& **B**); The effect of CCM@Ch-Cu/PtNPs on AGS and HGC for migration with a migratory percentage of cells the asterisk *indicates significance among groups (**C**-**D**)

JC-1 staining provided valuable insights into the mechanism of action of CCM@ChCu/PtNPs in inducing mitochondrial dysfunction and apoptosis in gastric cancer cells. These findings highlight the potential of CCM@ChCu/PtNPs as a promising therapeutic strategy for gastric cancer treatment, specifically through mitochondrial-targeted pathways shown in Sup Fig. 3. The results showed that miR-370 downregulation promoted GC cell motility and proliferation, in contrast to overexpression, which had the reverse effect [26]. The effects of CCM@Ch-Cu/PtNPs on a wound scratch test, which involved treating AGS and HGC cells with different concentrations for 6 and 12 h and then determining the relative wound width. This research has demonstrated that CCM@Ch-Cu/PtNPs can influence AGS and HGC migration rates (Fig. 10). Similarly, the effective natural antigen that can boost BMDC migration and adding TAAs to the mix even further enhances BMDC migration. Based on these results, it is reasonable to assume that DCs will migrate to lymph nodes and trigger T cell immunity In vivo [43].

### In vivo evaluation of CCM@Ch-Cu/PtNPs in xenograft gastric cancer model

**Fig. 11:**
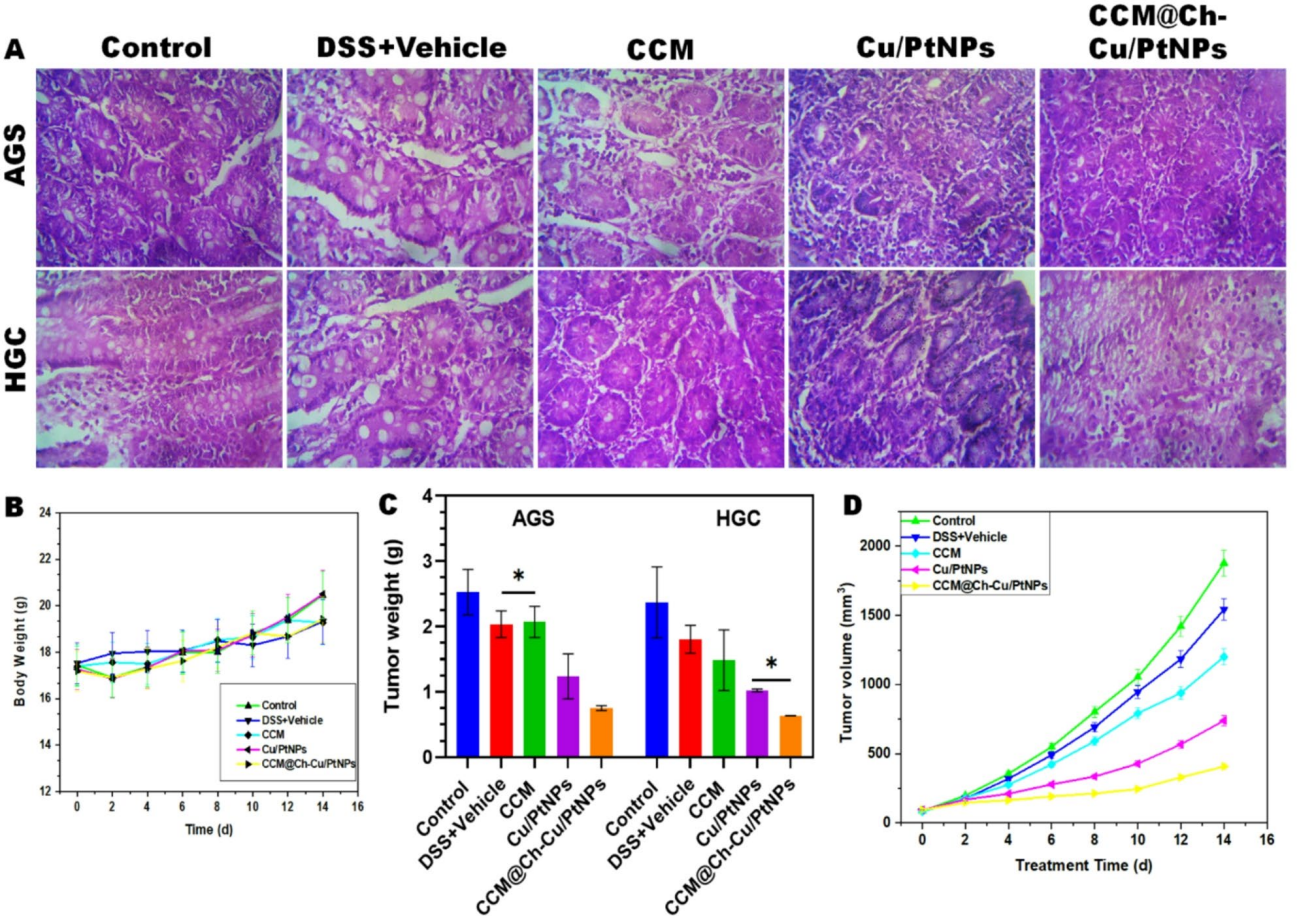
Histopathological analysis of gastric tissue treated with different samples for AGS and HGC xenograft bearing mice with 100 μm (**A**); The quantification analysis was done through body weight (**B**); Tumor weight (**C**); and tumor volume (**D**) with statistical significance

Tumor tissues removed from mice containing AGS and HGC xenografts underwent histological assessment subsequent to treatment with various formulations. Histological analysis (H&E staining) demonstrated substantial differences among the experimental groups (Fig. 11). Control tumor portions exhibited many tumor cells characterized by elevated nuclear-to-cytoplasmic ratios and disorganized structures, indicative of aggressive tumor proliferation. The DSS + Vehicle group showed more inflammatory infiltration and a lot of tumor growth, which shows that inflammatory stress can make tumors grow without treatment. Only treating with CCM caused a small improvement in histology, with a small decrease in tumor cell density; nonetheless, structural problems were still clear. The Cu/PtNP-treated group showed a big drop in tumor cellularity and a better organization of the tissue, which means that it was very effective against cancer. The group that had CCM@Ch-Cu/PtNP had the most obvious therapeutic benefit, which included a big drop in tumor cells, a rise in apoptotic morphology, a drop in mitotic figures, and a partial restoration of tissue architecture. Tumor development was substantially less than in other treatment groups. Over 16 days, body weight was checked to see how poisonous the system was. The CCM@Ch-Cu/PtNP group did not lose a lot of weight, and their numbers were about the same as those in the control group. This implies that the material poses minimal risk to the body and is compatible with living organisms. Quantitative data indicated that CCM@Ch-Cu/PtNP treatment markedly diminished tumor weight in comparison to DSS + Vehicle and other treatment cohorts (*p* < 0.05). Likewise, tumor growth curves indicated continuous tumor enlargement in the DSS + Vehicle group, while CCM@Ch-Cu/PtNP treatment produced the most significant reduction in tumor volume over time (*p* < 0.01). The glandular gastric mucosa of control mice showed no histological changes in contrast, animals exposed to carcinogens showed a higher incidence of neoplastic lesions [44]. Additionally, the mouse models allow for the first time In vivo testing of drug effects in systems that closely resemble human clinical trials for several types of stomach cancer. The well-established cancer organoid lines provide a high-tech, user-friendly In vitro tool for studying signaling pathways in cancer and screening possible targeted therapeutics on a broad scale [45, 46].

The histological examination of the main organs demonstrated that treatment with CCM@Ch-Cu/PtNPs not only displayed significant anti-gastric cancer efficacy but also reduced systemic toxicity. The tissues from this treatment group exhibited nearly normal histology in all assessed organs, underscoring its safety and therapeutic effectiveness. The DSS+Vehicle group exhibited considerable toxicity in all organs, whereas the CCM and Cu/PtNPs groups provided moderate protection, with the latter demonstrating greater efficacy than CCM alone. These findings highlight the promise of CCM@Ch-Cu/PtNPs as a secure and efficacious therapeutic approach for gastric cancer (Sup Fig. 4). Similarly, AT13148 treatment significantly reduced the volumes of HGC27 tumors in mice compared to vehicle control mice. AT13148 worked better at stopping HGC27 xenografts at a dose of 45 mg/kg per day than at 15 mg/kg, showing that the effect depends on the dose [47]. The evaluation of the antitumor efficacy of cetuximab, carboplatin, and their combination on tumor proliferation and metastasis in orthotopic gastric cancer xenografts in mice. This assessment included measuring the primary tumor volume and the metastatic spread of MKN-45 cells [48].

## Conclusion

In this study, we developed a biomimetic nanoplatform of cancer cell membrane-coated chitosan-Cu/Pt nanoparticles (CCM@Ch-Cu/PtNPs) that demonstrated a dual therapeutic and diagnostic potential for gastric cancer. The structural characterization confirmed the bimetallic nature of the nanoparticles, with a Cu-rich and Pt-rich surface, ensuring enhanced stability and functionality. In vitro experiments revealed that CCM@Ch-Cu/PtNPs effectively suppressed gastric cancer cell proliferation, induced apoptosis, and generated ROS, highlighting their ability to disrupt critical cellular pathways. Importantly, In vivo studies in a gastric cancer mice model showed selective tumor accumulation, significant tumor growth inhibition, and prolonged survival without observable toxicity to major organs. These findings highlight the potential of CCM@Ch-Cu/PtNPs as a promising nanoplatform for cancer-selective therapy, combining targeted treatment and diagnostic capabilities. This approach offers a step forward in the development of more effective and safer therapeutic strategies for gastric cancer, addressing the urgent need for precision medicine in oncology. Further studies on clinical translation will help validate the full potential of this innovative nanoplatform.

## Supplementary Information

Below is the link to the electronic supplementary material.

Supplementary Material 1

## Data Availability

Data will be made available on request. All the data generated or analysed during the study are included in this published article and its supplementary information files.

## Declarations

### aaaa

aaaa
