# Supplementary material for: Catalytic nanotherapeutics with cancer cell membrane and chitosan-coated Cu/Pt nanoparticles for gastric cancer precision therapy
Source: J Biol Eng. 2026 May 9;20:111. doi: 10.1186/s13036-026-00676-3 (PMC13326463; doi:10.1186/s13036-026-00676-3)

Sup. Fig 1: Nanoparticle formulation cumulative medication release profiles. CCM@Ch-Cu/PtNPs (cancer cell membrane-coated chitosan-Cu/Pt nanoparticles), Cu/PtNPs, and Ch (chitosan only) are compared for drug release in physiological settings. At 72 hours, CCM@Ch-Cu/PtNPs released the most drug (~90%), followed by Cu/PtNPs (~60%) and Ch alone (<20%). The biomimetic formulation exhibits enhanced drug release capabilities, as shown by mean ± standard deviation (n = 3).

Sup.Fig.2: CO conversion to CO2 elevated temperatures was used to investigate small and large Cu/Pt particles, The large and small particles after oxidation (Black), and reduction (Red) were observed. Then Cu particle re-reduction (pink) was analyzed.


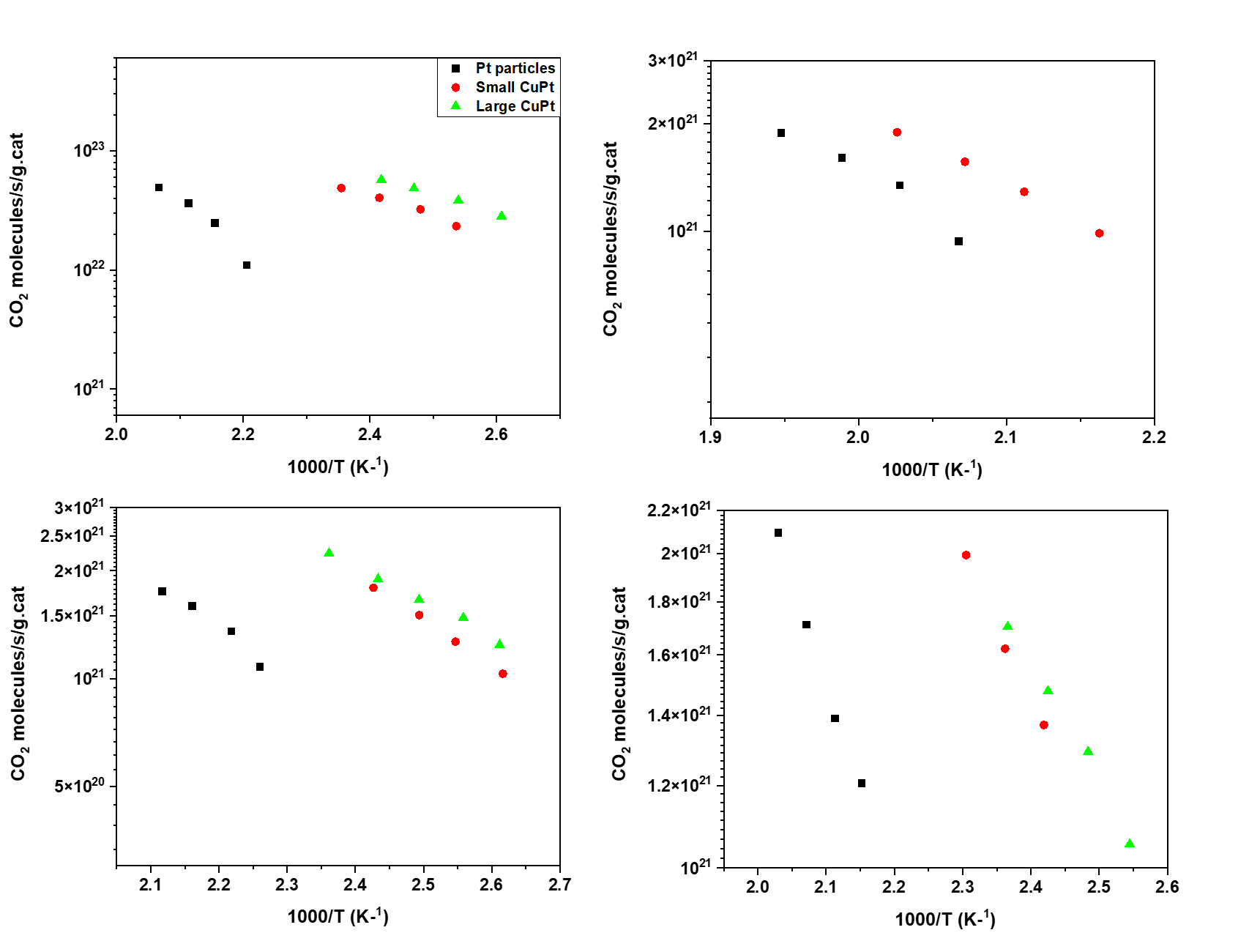


Sup.Fig.3: The ability of CCM@ChCu/PtNPs on AGS and HGC cell lines stained with JC-1 stain. Luminescent ATP levels in AGS (top) and HGC (bottom) after 24 h exposure to indicated formulations. Values normalized to vehicle (100%). Data = mean ± SD (n = 3).


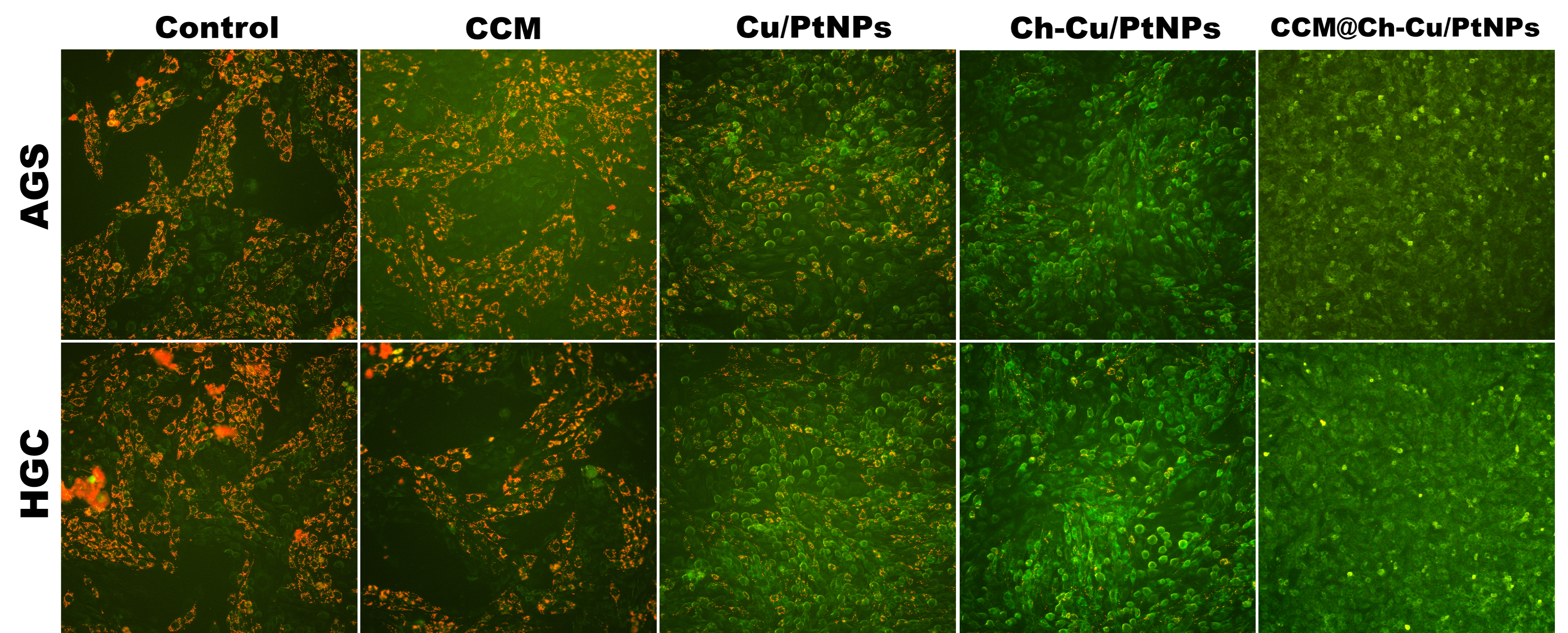


Sup.Fig.4: The histological observation on gastric cancer-induced mice showed fewer toxic effects on major organs after being treated with different samples.


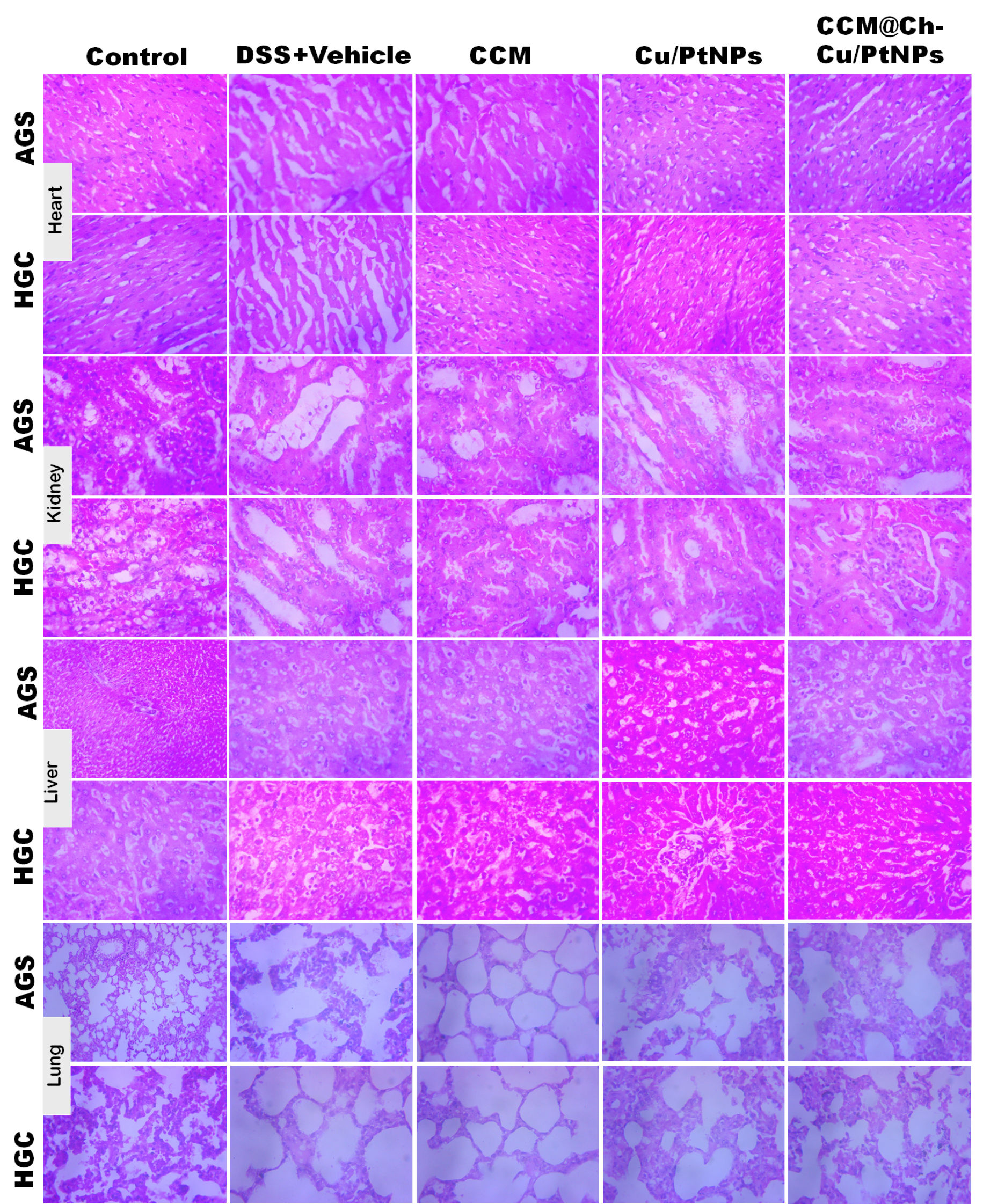

Supplement: Supplementary file 1 — Supplementary Material 1 [file 13036_2026_676_MOESM1_ESM.doc]
